# Scalable Multifunctional Fabrics with Boosted Intrinsic Photothermal Efficiency for Salt‐Resistant Solar‐Driven Janus Evaporators

**DOI:** 10.1002/advs.202504100

**Published:** 2025-05-29

**Authors:** Mingqing Yu, Jiaqi Hu, Xinghao Li, Linchu Xu, Huawei Hu, Robert T. Woodward, Wei Lyu, Yaozu Liao

**Affiliations:** ^1^ State Key Laboratory of Advanced Fiber Materials College of Materials Science and Engineering Donghua University Shanghai 201620 China; ^2^ Institute of Materials Chemistry and Research Faculty of Chemistry University of Vienna Währinger Straße 42 Vienna 1090 Austria

**Keywords:** confined polymerization strategy, hydrophobic fabrics, photothermal conversion, salt‐rejection, solar‐driven Janus evaporators

## Abstract

Global population growth and climate change are causing freshwater scarcity, which necessitates creative solutions like solar‐driven desalination. This technology's widespread adoption is hampered by cost and efficiency issues. Intrinsic photothermal conversion efficiency has gotten less attention than light trapping and thermal management, which have been the main focuses of efforts to increase photo‐to‐vapor efficiencies. Here, a commercial padding and vapor polymerization method are used to develop scalable nylon fabrics that act as solar absorbers. This is achieved by anchoring iron catalysts with hydrolyzed perfluorooctyltriethoxysilane chains, which cause confined polymerization of pyrrole to generate polypyrrole. By narrowing the bandgap and generating bioinspired light‐trapping nanostructures, this technique achieves a superior intrinsic photothermal conversion efficiency of 84.6%, which is 4.94 times higher than that of unconfined polymerization. These fabrics are used to create a Janus evaporator, which operates steadily in prolonged seawater testing and shows an evaporation rate of 3.84 kg m^−2^ h^−1^. The low manufacturing cost of ≈28 RMB m^−2^ emphasizes its scalability and economic potential. This work offers insights into the design of high‐performance, scalable, and cost‐effective solar absorbers by prioritizing increases in light absorption and intrinsic photothermal conversion efficiencies for developing solar desalination technology.

## Graphical abstract

Scalable nylon fabrics with bioinspired light‐trapping nanostructures and 84.6% intrinsic photothermal conversion efficiency are developed for solar desalination. The Janus evaporator shows high evaporation rate of 3.84 kg m^−2^ h^−1^ and long‐term stability in seawater, offering a cost‐effective solution for solar desalination. This material also emphasizes scalability and economic feasibility, making it suitable for widespread application.

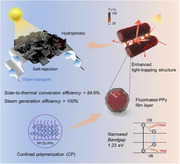

## 1 Introduction

Global population growth and climate change‐induced warming have exacerbated freshwater scarcity worldwide.^[^ ^1^ ^]^ Solar‐driven desalination, leveraging abundant renewable energy, has emerged as a promising solution, gaining significant attention in recent years.^[^ ^2^, ^3^ ^]^ Despite intensive efforts in materials and structural designs, such as light trapping, thermal management, and water supply, having been made to achieve thermodynamic‐limited solar‐to‐vapor efficiencies,^[^ ^4^, ^5^, ^6^, ^7^ ^]^ the crucial role of intrinsic photothermal conversion efficiency has received comparatively less attention. This efficiency, which governs the conversion of absorbed light into heat and its distribution within the material, is a pivotal second step following light absorption and preceding the phase‐change process in solar‐driven desalination. Beyond efficiency, large‐scale deployment of this technology faces economic constraints.^[^ ^8^, ^9^ ^]^ Progress in the scalable and cost‐effective fabrication of solar absorbers with enhanced intrinsic photothermal efficiency has been slow.^[^ ^10^ ^]^ This is largely due to challenges associated with widely studied active absorber materials: carbon‐based materials suffer from high thermal conductivity leading to heat loss, wide‐bandgap semiconductors exhibit poor infrared absorption, and powdery inorganic nanoparticles lack adequate film‐forming ability for scalable application.^[^ ^11^, ^12^ ^]^ Therefore, the scalable development of functional materials with high intrinsic photothermal conversion efficiency remains crucial for advancing solar‐driven desalination technology.

Bandgap engineering in semiconductors has proven to be an effective strategy for extending absorption across the spectrum and improving intrinsic photothermal efficiency by reducing heat emissions.^[^ ^13^, ^14^, ^15^, ^16^ ^]^ From an external perspective, combining bandgap engineering with light‐trapping nanostructures is expected to provide additional advantages by seamlessly integrating the critical first step of light absorption and the subsequent step of photothermal conversion in solar‐driven desalination technology. Specifically, hierarchical nanostructures with aligned arrays can trap light through multiple internal reflections, minimizing overall light reflections and improving light absorption efficiency.^[^ ^17^, ^18^, ^19^ ^]^ For example, black‐winged butterflies harness sunlight within their wing scales, trapping the light for functions such as de‐icing in cold weather.^[^ ^20^, ^21^, ^22^ ^]^ However, achieving scalable and precise bandgap tuning in such hierarchical micro‐ and nanostructured semiconductors through cost‐effective methods remains a significant challenge.

**Figure 1:**
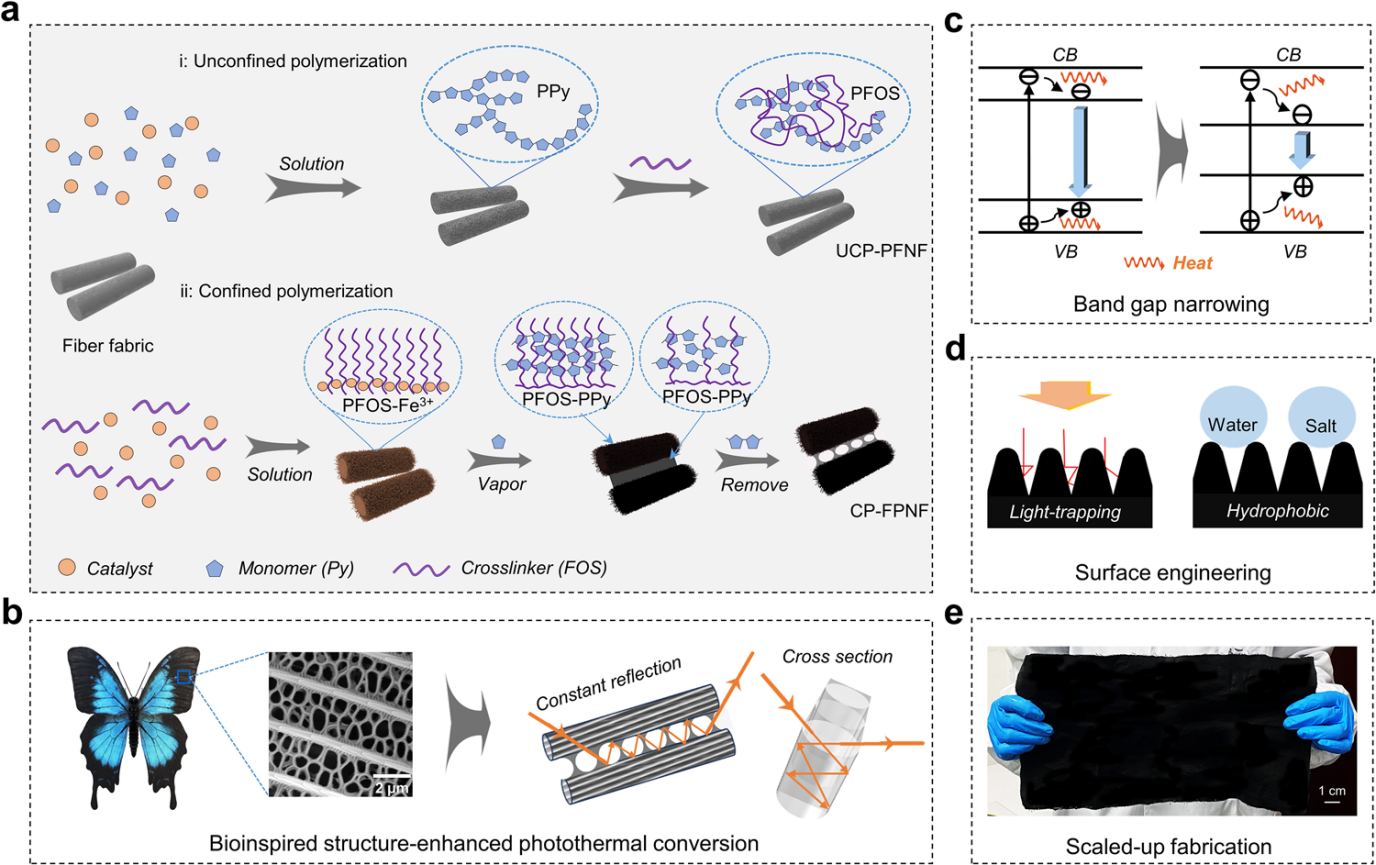
Schematic overview of the merits of fabric solar absorbers using a CP strategy. a) Comparison of fabric solar absorbers production using a UCP or CP strategy. b) Energy diagram showing the changes in the material's conduction band (CB) and valence band (VB). c) Scheme illustrating the enhanced solar absorption mechanism of the bioinspired light‐trapping nanostructure. d) Scheme illustrating the light‐trapping structures for enhanced external photothermal conversion and hydrophobic surface engineering for enhanced solar absorption and salt rejection. e) Photograph showing a scaled‐up CP‐based fabric.

In this study, scalable multifunctional nylon fabrics were developed as efficient solar absorbers by integrating commercial padding with vapor polymerization, resulting in both bioinspired light‐trapping nanostructures and narrowed bandgaps. Specifically, we employed a confined polymerization (CP) process, wherein iron catalysts were anchored using hydrolyzed 1H,1H,2H,2H‐perfluorooctyltriethoxysilane (FOS) chains pre‐coated on the fabrics. This process subsequently triggered pyrrole (Py) polymerization within the polyFOS (PFOS) network to form polypyrrole (PPy) (**Figure** **1**a). This approach endows fabrics with bioinspired, butterfly‐wing‐like light‐capturing nanostructures (Figure 1b), ensuring a high light absorption efficiency of 97.2%. Furthermore, this approach enables precise control over the material's bandgap (Figure 1c), allowing efficient light absorption across a broad spectrum and achieving an enhanced internal photothermal conversion efficiency (84.6%), which is 4.94 times higher than that of the unconfined polymerization (UCP) sample. Additionally, this method imparts fabrics with durable roughness and a hydrophobic surface (Figure 1d), ensuring long‐term stability while preventing salt deposition during operation. As a result, the Janus evaporator fabricated based on this CP‐based fabric demonstrated a significantly improved evaporation rate of 3.84 kg m^−2^ h^−1^ (≈245% higher than that of the UCP‐based fabric). No significant salt deposition was observed after 6 days of seawater testing. It is worth noting that the cost of this method is approximately 28 RMB m^−2^ (detailed evaluation provided in the Supporting Information), highlighting its affordability, scalability, and promising commercial potential (Figure 1e). This work highlights the integration of light absorption and photothermal conversion for advancing solar desalination technologies.

## 2 Results and Discussion

### 2.1 Preparation and Characterization of Multifunctional Fabric

**Figure 2:**
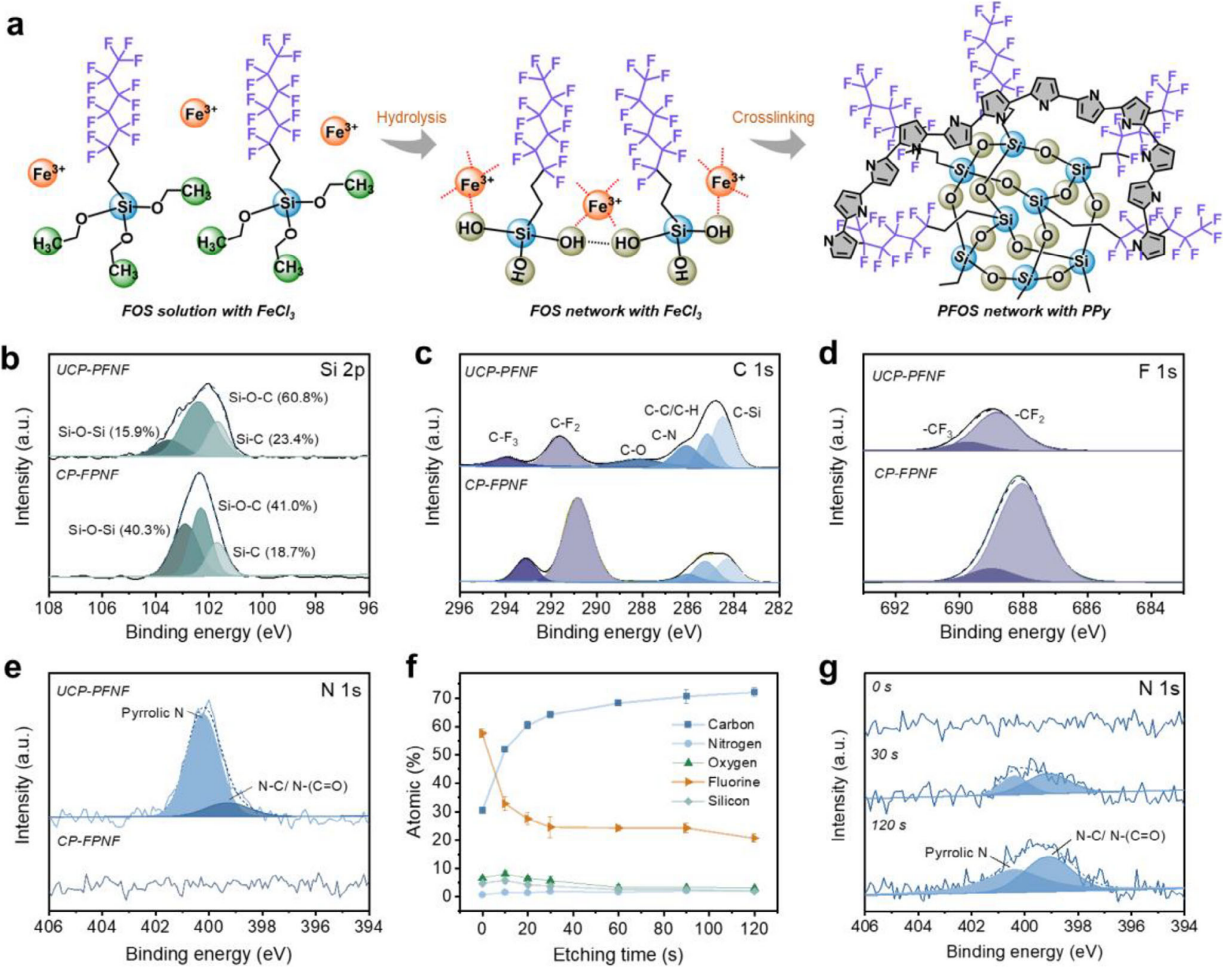
The fabrication and elemental composition of CP‐FPNF. a) Schematic illustration of the crosslinking process. b) Si 2p, c) C 1s, d) F 1s, and e) N 1s spectra of UCP‐PFNF and CP‐FPNF. f) Atomic content of CP‐FPNF with etching time (depth). Each value represents the mean ± standard deviation of three independent measurements. g) N 1s of CP‐FPNF at different etching times.

The fabrication of hydrophobic PFOS‐PPy/nylon fabric via a CP strategy (CP‐FPNF) is illustrated in **Figure** **2**a and Figure S1 (Supporting Information). Initially, dip‐coating was used to deposit FeCl_3_ catalyst and FOS uniformly onto the surface of a commercial nylon fabric. During this step, the triethoxysilane groups in FOS hydrolyzed into silanol groups (Si─OH), which bonded to the fiber surface through Si─OH···O═CNH─ hydrogen bonds and anchored Fe^3+^ catalysts via coordination interactions. Subsequently, Py was polymerized along the anchored Fe^3+^ sites, forming interactions with CF_2_ groups. Meanwhile, the upper FOS layer underwent self‐condensation and gradient crosslinking into PFOS via the Si─O─Si network during vapor polymerization, resulting in a rough film surface.^[^ ^23^ ^]^ For comparison, a control experiment using a conventional UCP method was conducted, where PPy was first polymerized and then coated with FOS. The resulting fabric was named PPy@PFOS/nylon fabric (UCP‐PFNF). The characteristic peaks for PPy's five‐membered ring stretching (1532 cm^−1^) and C─N─H group (1262 cm^−1^), along with peaks corresponding to the C─F bond (1146 and 1100 cm^−1^) from FOS,^[^ ^24^ ^]^ can be observed in the Fourier transform infrared (FT‐IR) spectra of CP‐FPNF (Figure S2, Supporting Information). Compared to UCP‐PFNF, the increased intensity of the Si─O─Si peak at ≈1018 cm^−1^ in the FT‐IR spectra of CP‐FPNF, as well as a higher percentage of Si─O─Si and a lower percentage of ─Si─O─C_2_H_5_ in the Si 2p X‐ray photoelectron spectroscopy (XPS) spectra (Figure 2b), suggest the self‐condensation of ─Si─O─C_2_H_5_ of FOS into PFOS.^[^ ^25^ ^]^ In the outermost C 1s XPS spectra (Figure 2c), lower binding energy peaks attributed to the ─CF_2_ (290.4 eV) and ─CF_3_ groups (292.9 eV) were observed.^[^ ^26^ ^]^ Similar shifts were noted for the F 1s XPS spectra (Figure 2d), reflecting differences in C─F bands, which may have resulted from the self‐condensation and gradient crosslinking. Peaks at 399.2 and 400.3 eV, corresponding to N─C/N─(C═O) and pyrrolic N, respectively,^[^ ^27^ ^]^ were absent in the N1s spectra of CP‐FPNF (Figure 2e).

Depth‐resolved XPS profiling supports the interpretation that PPy polymerization occurs within nano‐confined domains defined by a surface‐crosslinked PFOS layer (Figure 2f; Tables S1 and S2, Supporting Information). In the first 0–10 s of etching, the fluorine content decreases sharply from 57.52 ± 1.52% to 32.74 ± 1.42%, accompanied by a significant increase in carbon content. Meanwhile, nitrogen remains at a low level, indicating that the outermost region is composed primarily of fluorinated species, with minimal exposure of PPy. The simultaneous presence of silicon and oxygen suggests the formation of a chemically crosslinked Si─O─Si network at the surface, derived from the condensation of hydrolyzed PFOS molecules.^[^ ^25^ ^]^ Between 10 and 90 s, the gradual decrease in fluorine and silicon signals, together with a steady increase in nitrogen, reflect a compositional transition from the PFOS‐enriched surface toward a PPy‐dominated interior. The consistent detection of all elements throughout the depth profile implies a hierarchically crosslinked structure, in which PFOS and PPy are interpenetrated rather than sharply separated. This gradient architecture contrasts with conventional phase‐separated systems and reflects a continuous modulation in crosslinking density.^[^ ^28^ ^]^ At 120 s, a modest rise in nitrogen content is observed, which is likely attributable to the underlying nylon substrate that contains nitrogenous functionalities (Figure 2g). Altogether, the spatial evolution of elemental composition confirms that CP occurs within the vertically aligned fluoroalkyl domains formed by PFOS, enabling directional PPy growth and contributing to the structural order and performance of CP‐FPNF.

**Figure 3:**
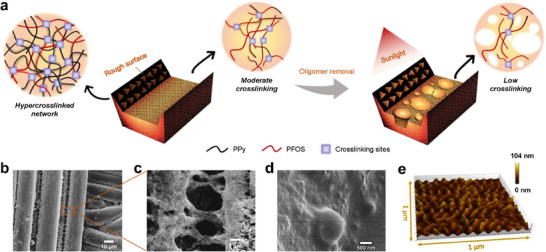
The bioinspired nanostructures of CP‐FPNF. a) Schematic representation of the formation of light‐trapping nanopore structures. SEM images of the CP‐FPNF with b,c) bioinspired butterfly wing morphology, and d) surface micro‐nanoroughness. e) AFM image of the CP‐FPNF fiber surface.

We then performed scanning electron microscopy (SEM) and atomic force microscopy (AFM) to characterize the proposed bioinspired micro‐ and nano‐structures. Oligomers between the fibers, which formed due to the absence of Fe^3+^ catalysts, were removed to develop bioinspired butterfly wing‐like light‐capturing nanostructures for efficient sunlight absorption (**Figure** **3**a; Figure S3, Supporting Information). The peaks observed in the ultraviolet–visible (UV–vis) spectra confirmed the presence of these oligomers (Figure S4, Supporting Information). SEM images shown in Figure 3b,c reveal the bioinspired nanoporous structure formed between parallel fibers. In contrast to the presence of only PPy particles on the surface of UCP‐PFNF fibers (Figure S5, Supporting Information), the fibrous surface of CP‐FPNF is characterized by PPy nanoparticles encapsulated within a PFOS film (Figure S6, Supporting Information). Additionally, the gradient crosslinking of FOS imparts a wrinkled surface to CP‐FPNF,^[^ ^23^, ^28^ ^]^ with a root mean square (RMS) roughness of 13.1 nm (Figure 3d,e). These bioinspired light‐trapping micro‐ and nanostructures are expected to significantly improve the light absorption efficiency of CP‐FPNF.

### 2.2 Bandgap Engineering via CP Strategy

**Figure 4:**
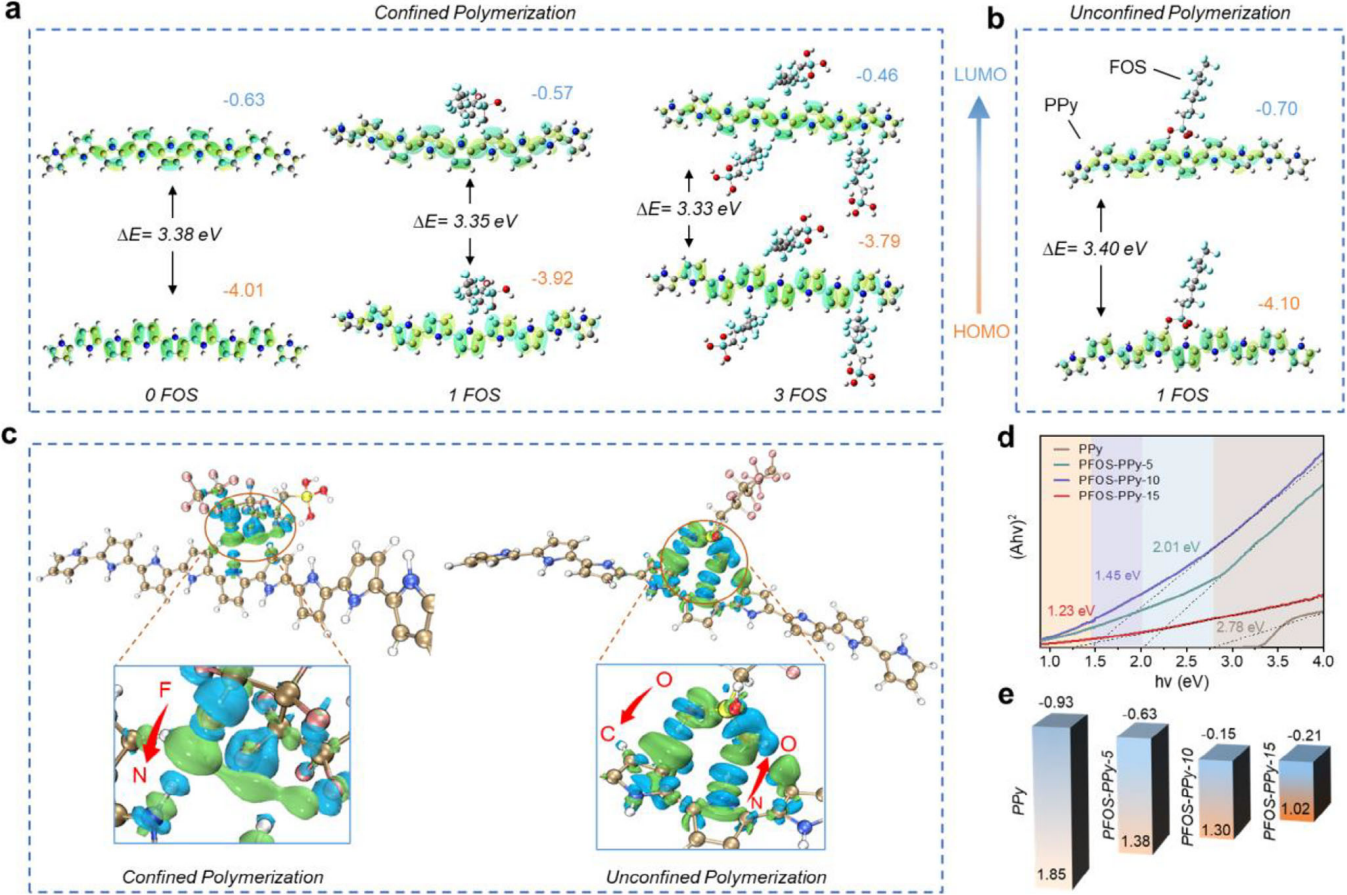
Merits of bandgap engineering via CP strategy. HOMO, LUMO, and bandgap of different PFOS‐PPy composite coatings, obtained via a) CP, and b) UCP strategy from DFT calculations. c) Charge transfer between PFOS and PPy, as calculated using DFT. d) Tauc's plots, and e) energy level diagrams were obtained from measurements.

When fluoroalkyl silanes are used as surfactant‐type additives in conventional polymerization systems, the molecules usually form micelle‐like structures with the hydrophilic Si─OH groups facing the PPy chains and the hydrophobic ─CF_2_/CF_3_ tails extending inward and remaining non‐interactive (Figure S7a, Supporting Information).^[^ ^29^ ^]^ In contrast, under CP conditions, the silanol groups undergo self‐condensation to form a Si─O─Si crosslinked network anchored on the fabric surface, which causes the fluoroalkyl chains to align more neatly (Figure S7b, Supporting Information). Unlike in conventional systems, the hydrophobic chains extend outward and guide PPy assembly under CP conditions, potentially enhancing charge delocalization and narrowing the bandgap of PPy. To gain further insight into the electronic structure of the samples and support the experimental findings, we conducted density functional theory (DFT) calculations on its energy band. As shown in **Figure** **4**a, confining PPy chains within PFOS results in narrower calculated bandgaps for PPy doped with one and three fluoroalkyl silane chains (FOS‐1: 3.35 eV, FOS‐3: 3.33 eV) compared to pure PPy (3.38 eV) and PPy doped with Si─OH groups (3.40 eV) (Figure 4b). Electron density difference (EDD) analysis (Figure 4c) reveals significant redistribution of electronic densities from the F atom of CF_2_ to the N atom of the Py ring, likely contributing to a higher conjugation and narrower bandgap in PFOS‐PPy. In contrast, unconfined PPy shows density redistribution from the N atom of the Py ring to the O atom of Si─OH groups.

A similar trend is observed in the Raman spectra of CP‐FPNFs with varied FOS content (CP‐FPNF‐0, CP‐FPNF‐5, CP‐FPNF‐10, and CP‐FPNF‐15), where the peak at 1556 cm^−1^, attributed to π conjugation (C─C) stretching vibrations, increases in intensity with higher F content (Figure S8, Supporting Information).^[^ ^30^ ^]^ To eliminate the influence of nylon fabric substrate, pure coatings (PPy, PFOS‐PPy‐5, PFOS‐PPy‐10, and PFOS‐PPy‐15) synthesized on glass slides using the same CP were analyzed for possible molecular interactions between PFOS and PPy. Notably, unlike the particle‐like structure of PFOS‐PPy polymerized in solution, the C P‐synthesized PFOS‐PPy coatings exhibit a film‐like structure similar to those on nylon fabric (Figure S9, Supporting Information). The increased intensity of peaks around 1556 cm^−1^ in the FT‐IR spectra (Figure S10, Supporting Information) of PFOS‐PPy‐10 and PFOS‐PPy‐15 indicate a higher degree of conjugation in the ordered PPy chains within the composite materials.^[^ ^31^ ^]^ Additionally, the gradually decreased intensity of the peak at 3433 cm^−1^ (N─H bond) and the emergence of a peak at 1193 cm^−1^, characteristic of PPy doping,^[^ ^32^ ^]^ verify the doping effect of PFOS. Combined with the DFT calculation, we can deduce that this doping resulted from the ─CF_2_ group. The narrowed bandgap measured from the Tauc plot with increasing fluorine content in PFOS‐PPy composites further supports the occurrence of the tunable bandgap of the CP method (Figure 4d),^[^ ^33^ ^]^ with 2.78, 2.01, 1.45, and 1.2 eV calculated for PPy, PFOS‐PPy‐5, PFOS‐PPy‐10, and PFOS‐PPy‐15, respectively. Combined with the measured Mott‐Schottky curve (Figure S11, Supporting Information),^[^ ^34^ ^]^ the detailed conduction band and valence band potentials can be seen in Figure 4e.

### 2.3 Integrated Photothermal and Vapor Conversion

**Figure 5:**
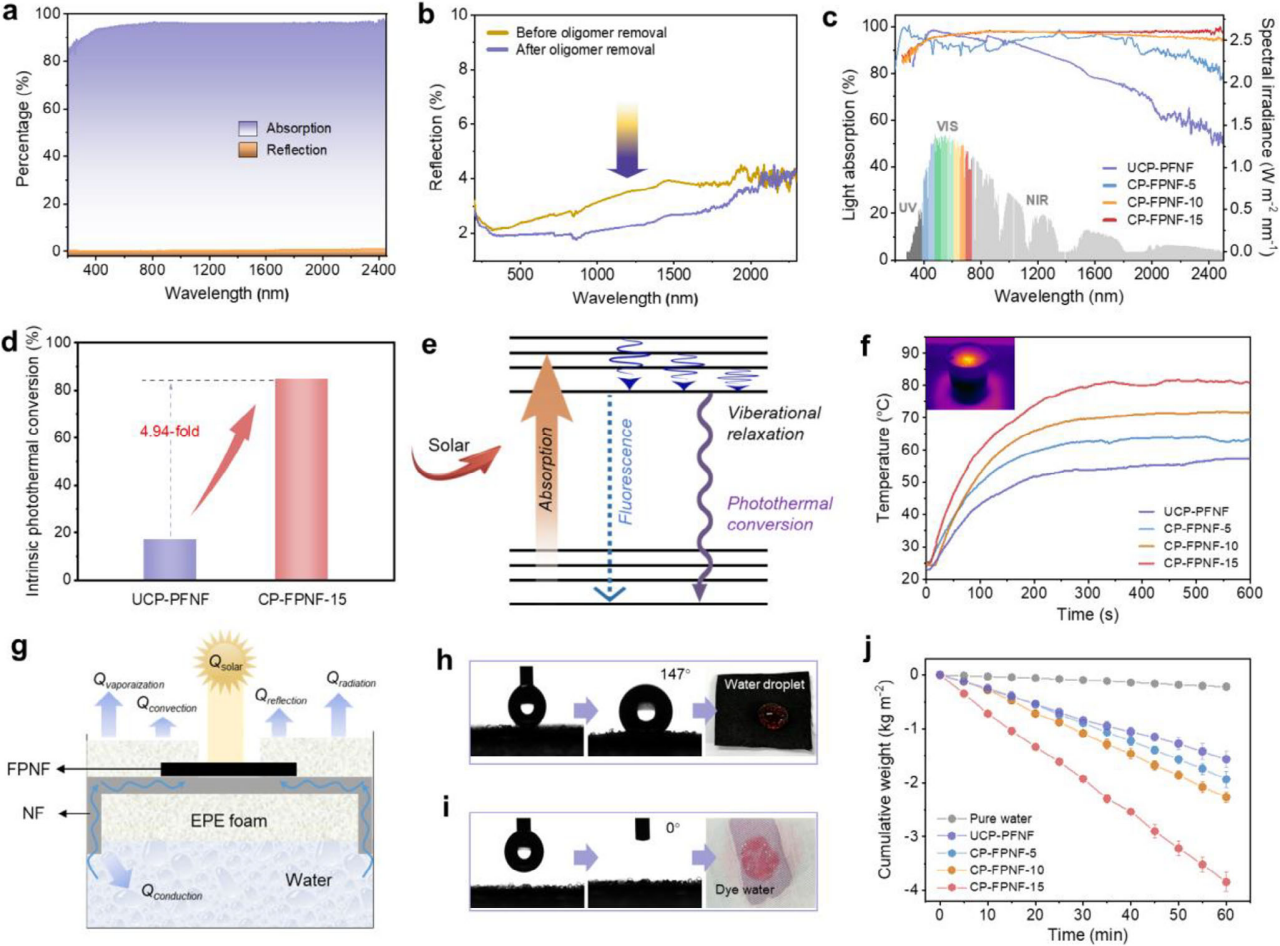
a) UV–Vis–NIR absorption and reflectance spectra of CP‐FPNF‐15. b) Reflectance spectra of CP‐FPNF‐15 before and after oligomer removal. c) Absorption spectra of the UCP‐PFNF and CP‐FPNFs. d) Photothermal conversion efficiency of UCP‐PFNF and CP‐FPNF‐15. e) Schematic of photothermal conversion, focusing on electronic transitions and energy loss pathways under optical excitation. f) Surface temperature variation of evaporators over time under 1 sun irradiation. g) Schematic of photo‐to‐vapor conversion in the Janus evaporator device, illustrating energy utilization. h) Contact angles and photograph of the top hydrophobic layer. i) Contact angles and photograph of the bottom hydrophilic layer. j) Mass variation of water, UCP‐PFNF, and CP‐FPNF‐15 under 1 sun irradiation. Each value represents the mean ± standard deviation of three independent measurements.

As expected, an ultrahigh absorption efficiency of 97.2% is achieved for CP‐FPNF‐15, along with a reflectance of 0.3% across the entire spectrum (200–2500 nm) (**Figure** **5**a). This absorption efficiency is due to the multiple light reflections within nanoholes from its bioinspired light‐trapping nanostructure and the increased area for light‐trapping from its wrinkled surface. The higher reflectance of CP‐FPNF without oligomer removal verifies the contribution of the bioinspired light‐trapping nanostructure to the reduced light reflection (Figure 5b). As shown in Figure 5c, CP‐FPNFs exhibit similar absorption values of 92.2%, 95.1%, and 97.2% for CP‐FPNF‐5, CP‐FPNF‐10, and CP‐FPNF‐15, respectively, which is much higher than that of UCP‐PFNF (80.6%). Encouraged by the high absorption values, the intrinsic photothermal efficiency (*η*) of the materials was further evaluated using the cooling‐curve method under 1 sun irradiation (Figure S12; Equations S1–S8, Supporting Information).^[^ ^35^, ^36^ ^]^ This method calculates *η* based on the thermal energy stored during irradiation and the heat dissipation rate during cooling. As shown in Figure 5d, CP‐FPNF‐15 achieves an intrinsic photothermal efficiency of 84.6%, significantly higher than that of UCP‐PFNF (17.1%). CP‐FPNF‐5 and CP‐FPNF‐10 obtain an intrinsic photothermal efficiency of 52.4% and 67.6%, respectively (Figure S13, Supporting Information). This enhanced efficiency is attributed to the increased electronic conjugation and reduced bandgap in PPy, which arise from the incorporation of fluoroalkyl chains during CP. As illustrated in Figure 5e, photons with energy above the bandgap excite electron–hole pairs. In narrow bandgap materials, these carriers undergo non‐radiative relaxation, converting excess energy into heat. In contrast, wider bandgap materials tend to dissipate energy via radiative recombination (fluorescence).^[^ ^37^, ^38^ ^]^ The tailored band structure in CP‐FPNF‐15 facilitates efficient light‐to‐heat conversion at the molecular level, underpinning its high intrinsic *η* value. The fluorescence spectra of the samples (Figure S14, Supporting Information) show a clear decrease in photoluminescence (PL) intensity with increasing FOS content, particularly in CP‐FPNF‐15. This suggests a non‐radiative decay processes, indicating a narrowing of the bandgap, which is further supported by a shift in the PL peak to longer wavelengths.^[^ ^39^ ^]^ These changes align with the DFT calculations, which also predict a bandgap reduction with increasing FOS concentration. These combined features, including efficient non‐radiative decay and extended absorption, contribute to the superior photothermal conversion performance of CP‐FPNF, as confirmed by the measured efficiency.

As shown in Figure 5f, UCP‐PFNF reached a plateau at 50 °C, while CP‐FPNF‐15 reached 80 °C within 5 minutes under 1 sun irradiation. The photothermal stability of CP‐FPNF‐15 was further assessed by repeated light on and off cycles (Figure S15, Supporting Information). Across six consecutive cycles, the material exhibited fast thermal response with no degradation in peak temperature or heating rate, demonstrating its excellent short‐term photothermal stability. As shown in Figure S16 (Supporting Information), the SEM image of the fiber surface after cycling remains nearly unchanged when compared to the pristine state, with no observable cracks, delamination, or any sign of degradation. This result confirms the excellent structural robustness of the polymer layer under repeated thermal stress, supporting its reliability in practical applications. To achieve a highly efficient interfacial evaporator, we designed a CP‐FPNF‐based Janus structure^[^ ^40^ ^]^ integrated with a 2D water channel (Figure 5g). The hydrophobic CP‐FPNF‐15 surface (contact angle: 147°) minimizes heat loss and salt scaling, while the hydrophilic nylon substrate facilitates continuous water delivery (Figure 5h,i; Figure S17, Supporting Information). This asymmetric wettability, combined with the use of low‐thermal‐conductivity polyethylene foam layers on both sides of the fabric, creates a thermally confined and water‐managing structure conducive to efficient evaporation.

The CP‐FPNF‐15 evaporator achieved an outstanding evaporation rate of 3.84 kg m^−2^ h^−1^ under 1 sun irradiation, compared to 1.57 kg m^−2^ h^−1^ for UCP‐FPNF and only 0.25 kg m^−2^ h^−1^ for the control sample without any solar absorber (Figure 5j). Figures S18 and S19 (Supporting Information) show that the effective vaporization enthalpy of CP‐FPNF‐15 (1235 J g^−1^) is markedly lower than that of bulk water (2450 J g^−1^), likely due to confined water environments.^[^ ^41^, ^42^ ^]^ Thermal energy analysis (Equations S11–S13, Supporting Information) revealed that convective, radiative, and conductive heat losses together account for less than 5% of the incident energy under 1 sun irradiation, further enhancing photothermal performance.^[^ ^43^ ^]^ Notably, this rate exceeds the theoretical limit of 1.47 kg m^−2^ h^−1^ based on 100% solar‐to‐vapor efficiency, indicating that additional mechanisms are involved.^[^ ^44^, ^45^, ^46^ ^]^ This enhancement can be attributed to several synergistic effects: i) the 3D woven substrate and rough photothermal surface provide a large effective evaporation interface and enhanced light trapping; ii) the porous architecture and Janus design facilitate continuous water transport, which may reduce the effective vaporization enthalpy by weakening hydrogen bonding at the water‐solid interface; and iii) the hydrophobic surface and multilayer structure potentially enable partial reutilization of the latent heat from rising vapor, especially under confined conditions. These contributing factors have also been emphasized in recent studies on high‐performance solar evaporators​.^[^ ^47^ ^–^ ^49^ ^]^ To simulate practical desalination conditions, saline solutions with varying salt concentrations (0–26.4 wt%) were tested. The CP‐FPNF‐15‐based Janus evaporator maintained a stable evaporation rate of ≈2.00 kg m^−2^ h^−1^ even in saturated brine (Figure S20, Supporting Information), confirming its strong salt tolerance and application potential.

### 2.4 High‐Efficiency Evaporation and Contaminant Removal

**Figure 6:**
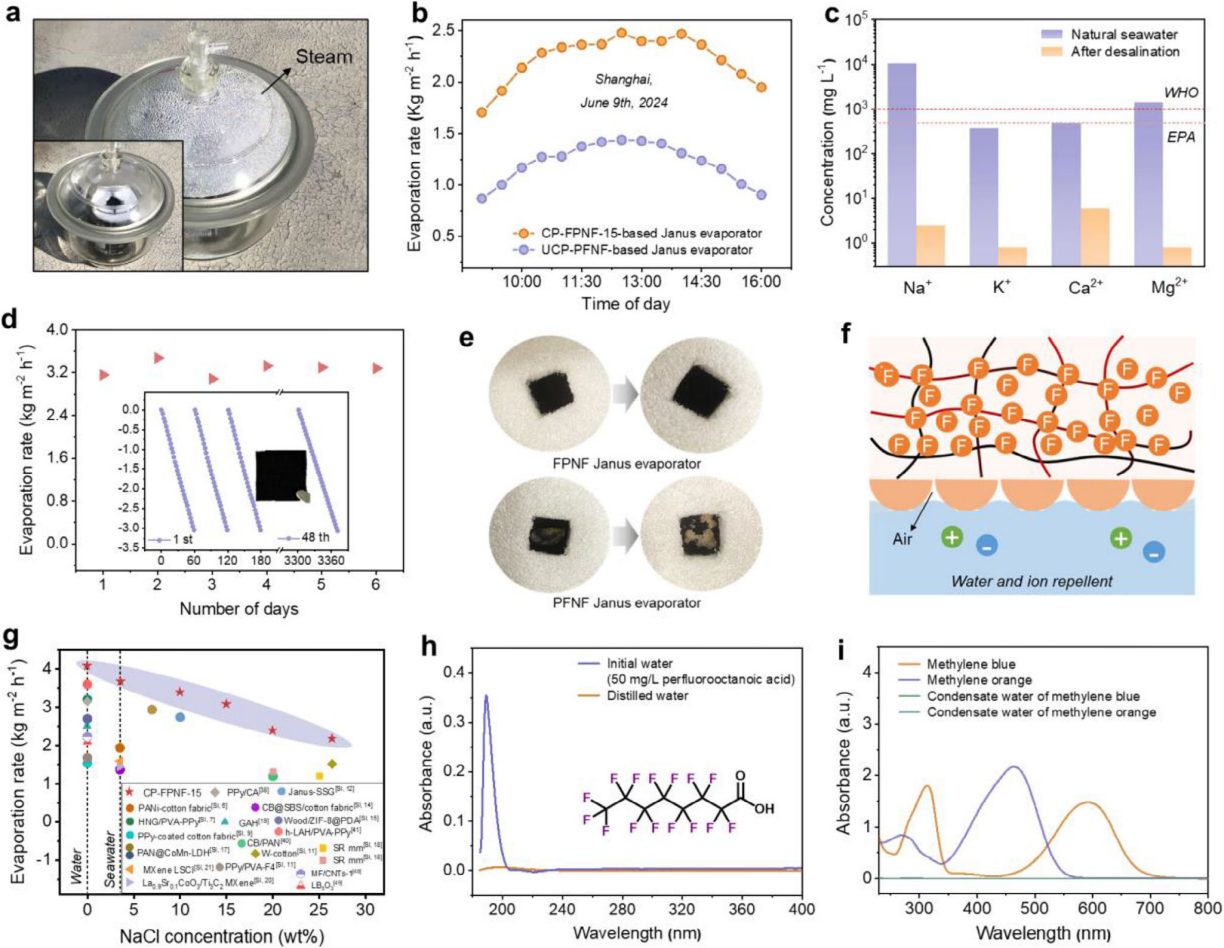
a) Photograph of the outdoor solar evaporation setup (inset: system before evaporation). b) Outdoor evaporation rate at different daylight hours. c) Ion concentrations in East China Seawater before and after desalination. d) Long‐term solar exposure test of CP‐FPNF‐15 under simulated saltwater evaporation conditions (1 sun irradiation, 40% humidity, 8 h per day exposure). e) Images of the evaporators' surfaces following 2000 min of constant solar evaporation with seawater. f) Mechanism of ion trapping between the CP‐FPNF‐15 and seawater. g) Comparison of the CP‐FPNF‐15‐based Janus evaporator evaporation rates with those in other reported literature. h) Removal of PFOA contaminants by the CP‐FPNF‐15‐based Janus evaporator. i) Removal of MB and MO contaminants by the CP‐FPNF‐15‐based Janus evaporator.

Building on the promising results of small‐scale samples, a large‐area (22 × 16 cm^2^) CP‐FPNF‐15 was prepared to evaluate the scalability and real‐world applicability of the CP strategy. To further examine the consistency of this scalable fabrication, we tested three independent batches of CP‐FPNF‐15. All samples exhibited comparable average evaporation rates, with only minor temporal fluctuations (Figure S21, Supporting Information). Infrared thermal imaging further confirmed uniform photothermal performance across the large‐area fabric, indicating excellent scalability and consistency (Figure S22, Supporting Information). These results demonstrate the robustness and scalability of the CP method without the need for additional templates or surface patterning. We then constructed an outdoor setup (**Figure** **6**a) and evaluated the solar evaporation performance in Songjiang, Shanghai, on a clear day. Figure 6b shows the cumulative mass loss of a 10% NaCl solution measured at 30 min intervals under varying solar irradiance. At peak sunlight, the CP‐FPNF‐15‐based Janus evaporator achieved an evaporation rate of 2.49 kg m^−2^ h^−1^, nearly double that of the UCP‐PFNF control. It is worth noting that outdoor evaporation rates were slightly lower than those measured indoors, likely due to factors such as non‐vertical solar incidence, fluctuating irradiance, and higher ambient humidity. In contrast, indoor testing under stable illumination and controlled humidity (Figure S23, Supporting Information) provided a more consistent environment, leading to higher and more stable evaporation rates.^[^ ^50^, ^51^ ^]^

Seawater from the East China Sea was used for desalination experiments to validate the real‐world feasibility. The quality of the purified water was analyzed using inductively coupled plasma optical emission spectrometry. As shown in Figure 6c, the concentrations of four major cations (Na^+^, K^+^, Mg^2+^, Ca^2+^) in the desalinated water were significantly reduced and met the World Health Organization (WHO) drinking water standards. Additionally, the electrical resistance of the condensed water (1.1 MΩ) was much higher than that of the raw seawater (403.1 KΩ), further confirming the effective purification process (Figure S24, Supporting Information). Moreover, long‐term stability tests were conducted to evaluate the durability of the CP‐FPNF‐15‐based Janus evaporator under continuous simulated solar irradiation. We conducted a six‐day continuous solar‐driven evaporation test using 3.5% NaCl solution under 1 sun irradiation (8 h per day, 40% RH). As shown in Figure 6d, the evaporator maintained a stable and high evaporation rate throughout the entire testing period, with consistent hourly mass loss. Notably, after six days of operation, no visible salt crystallization was observed on the evaporator surface (inset photograph), indicating excellent salt‐resistance and structural integrity. In contrast, UCP‐FPNF‐based evaporators exhibited significant salt accumulation under similar conditions, which impaired light absorption and hindered steam generation (Figure 6e). The superior performance of CP‐FPNF‐15 is attributed to its hierarchical micro/nanostructure and low surface energy, which collectively enable efficient water transport while preventing salt backflow and deposition (Figure 6f).^[^ ^52^ ^]^

We performed manual folding and rubbing experiments to further evaluate the CP‐FPNF‐based Janus evaporator's resilience to mechanical stress. The evaporator maintained both structural integrity and stable evaporation performance after five cycles of mechanical treatment (Figure S25, Supporting Information). Furthermore, tensile testing revealed that the CP‐FPNF had substantially greater ductility (387% strain) than the UCP‐PFNF (193% strain), which is advantageous for withstanding mechanical deformations such as bending and flapping that occur outdoors (Figure S26, Supporting Information). Compared to advanced solar evaporators reported in the literature, the CP‐FPNF‐15‐based Janus evaporator demonstrated superior evaporation rates across a range of salt concentrations, as showcased in this study (Figure 6g; Table S3, Supporting Information). Additionally, the CP‐FPNF‐15‐based Janus evaporator can effectively remove perfluorooctanoic acid (PFOA), methylene blue (MB), and methyl orange (MO) from simulated contaminated water. As depicted in Figure 6h,i, the strong adsorption peaks observed in the original solutions of PFOA, MB, and MO disappeared completely after photothermal purification, achieving a removal efficiency exceeding 99%. The remarkable PFOA‐rejection capability arises from the micro‐nanostructure and hydrophobic surface of CP‐FPNF‐15, which facilitate the adsorption of PFOA molecules through hydrophobic interactions, as supported by previous studies.^[^ ^53^ ^]^ The above results verify that the CP‐FPNF‐15‐based Janus evaporator displays self‐cleaning and anti‐fouling properties, driven by solar energy while maintaining highly efficient photothermal water purification performance.

## 3 Conclusions

This study introduces a scalable and cost‐effective approach for the development of high‐performance solar absorbers by integrating bandgap engineering with bioinspired light‐trapping nanostructures. Using a CP process, nylon fabrics can be transformed into advanced solar absorbers with an exceptional intrinsic photothermal conversion efficiency of 84.6%. These attributes contributed to a remarkable evaporation rate of 3.84 kg m^−2^ h^−1^ in solar‐driven desalination applications, representing a significant enhancement over conventional methods. Additionally, the durable roughness and hydrophobic surface of the fabrics ensured long‐term operational stability, effectively preventing salt deposition during extended seawater testing. The low production cost of approximately 28 RMB m^−2^ further highlights the scalability and commercial potential of this approach. By addressing intrinsic photothermal efficiency alongside scalable material development, this work offers a meaningful contribution to the advancement of solar desalination technology and provides a viable pathway to alleviate global freshwater scarcity.

## 4 Experimental Section

### Materials

All aqueous solutions were prepared using deionized water, and all reagents were used as received. Py was purchased from Vanadium Technetium Technology Co., Ltd., Shanghai. FeCl_3_∙6H_2_O, NaCl, and FOS were bought from Macklin Biochemical Technology Co., Ltd., Shanghai. PFOA was purchased from Shanghai Merrell Chemical Technology Co., Ltd. MB and MO were purchased from Shanghai Aladdin Biochemical Technology Co., Ltd. Commercial polyamide 6 (nylon 6) fabric, glass microscope, and expanded polyurethane foam were commercially available. A Master‐S30 Hitech apparatus produced deionized water. Seawater was obtained from the beach on Gulangyu Island, Xiamen, China. Ethanol was purchased from Sinopharm Chemical Reagent Co.

### The Fabrication of CP‐FPNF

Initially, a precursor solution containing FOS (0–15 wt%) and FeCl_3_ (0.5 g) was prepared in ethanol (20 mL) and sonicated for 5 min to ensure homogeneity. The cleaned fabric was then immersed in the solution for 15 min, followed by drying, resulting in a fiber‐loaded catalyst and a weakly crosslinked PFOS layer. Py polymerization was selectively confined within the PFOS network on the fiber surface using Py vapor. Specifically, the fabric and 1 mL of Py monomer were placed in a sealed glass container, which was heated to 80 °C on a hot plate. Within minutes, vapor generation began, and the fabric turned completely black after 0.5 h, indicating the successful formation of PPy. The heating process was then stopped. Subsequently, the fabric was soaked in ethanol and subjected to ultrasonic stirring for 1 h to remove unreacted materials until the rinse solution became colorless. The fabric was then air‐dried overnight at room temperature. FPNF samples prepared with different FOS concentrations (0%, 5%, 10%, 15%) were labeled as CP‐FPNF‐0, CP‐FPNF‐5, CP‐FPNF‐10, and CP‐FPNF‐15, respectively. To investigate the properties of PFOS‐PPy composite layers, the same CP process was applied to glass slide substrates instead of fabrics. Samples obtained with varying FOS concentrations 0%, 5%, 10%, and 15% were labeled as PPy, PFOS‐PPy‐5, PFOS‐PPy‐10, and PFOS‐PPy‐15, respectively.

### The Fabrication of UCP‐PFNF

An ethanol solution (20 mL) containing FeCl_3_ (0.5 g) was sonicated for 5 min to ensure optimal homogeneity. The cleaned fabric was then immersed in the solution and dried for 15 min, resulting in the distribution of the FeCl_3_ catalyst on the fabric. Subsequently, a mixed solution of 1 mL Py monomer and water was added to a beaker, and the fabric was immersed and stirred at room temperature. During this process, the solution initially appeared yellow due to the presence of FeCl_3_ but gradually turned black, indicating that the FeCl_3_ catalyst was not fixed on the fabric. After 4 h of polymerization, the fabric turned black, confirming the formation of PPy, and the stirring was stopped. Finally, the PPy‐coated fabric was immersed in an ethanol solution containing 15% FOS for 0.5 h and then air‐dried overnight at room temperature. The resulting fabric, prepared using 15% FOS, is designated as UCP‐PFNF.

### Characterization

Field emission scanning electron microscopy (SEM, SU8010, HITACHI, Japan) was used to investigate the microstructure, and energy‐dispersive spectroscopy (EDS) mapping revealed the elemental distribution. Atomic force microscopy (AFM, Agilent 5500) was used to measure surface roughness. Static contact angle (CA) measurement was determined at room temperature using an OCA 40 Micro machine (Dataphysics, Germany) to characterize the amphiphilicity of the specimens. The average CA values were determined by measuring the same sample in 3–5 different positions. The volume of the water droplet was 3 µL for CA unless noted otherwise. The phase and crystal structure of prepared samples were examined by X‐ray diffraction (XRD) patterns using an X‐ray diffraction spectrometer (Rigaku, D/Max2550VB+/PC,18 KW, Cu Kα radiation source). Raman spectra were measured by Reflex laser Raman spectrometer. Fourier transform infrared spectroscopy (FTIR) of samples was obtained using a Nicolet 670 spectrometer. The X‐ray photoelectron spectroscopy (XPS) spectra were measured using a spectrometer (Escalab 250Xi). Diffuse reflectance UV–vis–NIR spectra were recorded on a UV‐2600i spectrometer (referenced to barium sulfate). The metal ion concentrations of the seawater and steam water were measured using an inductively coupled plasma‐optical emission spectrometer (ICP‐OES, PerkinElmer Optima 7300 V, USA). The steady‐state photoluminescence (PL) spectra were collected by QM/TM fluorometer with an excitation wavelength of 295 nm.

### Mott‐Schottky (MS) Measurements

The MS measurements were performed on an Interface 1000E (Germany) electrochemical workstation, using a standard three‐electrode system: 0.2 m Na_2_SO_4_ aqueous solution as an electrolyte, a platinum plate as the counter electrode, an Ag/AgCl electrode as the reference electrode, and a glassy‐carbon electrode coated with the sample as the working electrode. The working electrode was prepared as follows: 4 mg of powder sample was dispersed in a mixture of 1 mL methanol and 80 µL Nafion, sonicated to obtain a homogeneous dispersion, and 2 µL of dispersion was deposited onto a glass‐carbon electrode (0.07 cm^2^), and dried at room temperature for 60 min. The flat band potential of the material was obtained from the tangent of the longest linear segment of the MS curve and the intercept of the X‐axis, and the conduction band potential of the material was obtained using the following equation (1):

(1) $$E_{\mathrm{RHE}}=E\left(\mathrm{Ag}/\mathrm{AgCl}\right)+0.0591\mathrm{pH}+E_{\;\mathrm{Ag}/\mathrm{AgCl}}^{\theta }\left(E_{\;\mathrm{Ag}/\mathrm{AgCl}}^{\theta }=0.199\;V\right)$$

### Solar Evaporation Performance Testing

Solar evaporation performance was evaluated using a solar simulator (Solar‐500, Beijing NBeT Technology Co., Ltd, China) equipped with an AM 1.5 solar filter to generate radiation at a power density of 1 kW m^−2^ (equivalent to 1 sun). The evaporators were placed in beakers containing different water environments, with polyethylene foam (1 cm thick) serving as both upper and lower thermal insulation layers to wrap the solar collector and isolate it from the water. A small opening with a projected area of 1 cm^2^ was made at the center of the upper foam layer. The device was mounted on an electronic analytical balance for real‐time recording of water evaporation. Surface temperature data were captured using an infrared camera (FOTRIC 220s).

### Statistical Analysis

All experimental data were obtained from three independent measurements and are presented as mean ± standard deviation, as shown by the error bars in the corresponding figures. No statistical hypothesis testing was applied in this study. All data processing and plotting were performed using Origin software.

## Conflict of Interest

The authors declare no conflict of interest.

## Supporting information



Supporting Information

## Data Availability

The data that support the findings of this study are available from the corresponding author upon reasonable request.
