# Supplementary material for: Scalable Multifunctional Fabrics with Boosted Intrinsic Photothermal Efficiency for Salt‐Resistant Solar‐Driven Janus Evaporators
Source: Adv Sci (Weinh). 2025 May 29;12(30):e04100. doi: 10.1002/advs.202504100 (PMC12376551; doi:10.1002/advs.202504100)
Supplement: Supplementary file 1 — Supporting Information [file ADVS-12-e04100-s001.docx]

Supporting Information

**Scalable Multifunctional Fabrics with Boosted Intrinsic Photothermal Efficiency for Salt-Resistant Solar-Driven Janus Evaporators**

*Mingqing Yu,^1,2^ Jiaqi Hu,^1^ Xinghao Li,^1^ Linchu Xu,^1^ Huawei Hu,^1^ Robert T. Woodward,^2^* Wei Lyu,^1^* and Yaozu Liao^1^**

^1^State Key Laboratory of Advanced Fiber Materials, College of Materials Science and Engineering

Donghua University

Shanghai 201620, China

^2^Institute of Materials Chemistry and Research, Faculty of Chemistry

University of Vienna

Währinger Straße 42, Vienna 1090, Austria

*Corresponding author.

E-mail: robert.woodward@univie.ac.at; wlyu@dhu.edu.cn; yzliao@dhu.edu.cn

**Table of Contents**

1. Calculation section S1

1.1. Cost analysis of the fabrication method S1

1.2. Calculation of the photothermal conversion efficiency S3

1.3. Calculation of the photo-to-vapor conversion efficiency S5

1.4. Analysis of heat transfer mechanisms in the solar interfacial evaporation system S7

2. Figures (S1-S26) S9

2.1. Synthesis scheme of CP-FPNFs S9

2.2. Structure characterization of multifunction fabrics S10

2.3. Microscopic surface morphology of fibers S12

2.4. Microscopic surface morphology of PPy and PFOS-PPy coatings S14

2.5. Structure characterization of PPy and PFOS-PPy coatings S15

2.6. Solar evaporation performance testing S17

3. Tables (S1-S3) S22

4. Supplementary references S24

# 1. Calculation section

## 1.1. Cost analysis of the fabrication method

The calculation of the cost (27.53 RMB m^−2^) for the proposed fabrication method is based on the following components:

**Raw materials (CP-FPNF-15 as an example)**

1. 1H,1H,2H,2H-perfluorooctyltriethoxysilane (FOS): Each batch uses 0.78 mL of FOS to prepare a 10 mL ethanol solution. For 5 pieces of fabric (6 cm × 6 cm each), this corresponds to 43.33 mL m^−2^ of FOS solution. With a density of approximately 1.3 g mL^−1^, this equates to 56.33 g m^−2^ of FOS. The bulk purchase price for industrial-grade FOS (kilogram-level quantities) is approximately 2200 RMB kg^−1^, or 2.2 RMB g^−1^. Assuming the reuse of the solution 5 times, the effective cost is reduced to approximately 24.80 RMB m^−2^.
2. Iron (III) chloride hexahydrate (FeCl_3_ 6H_2_O): Each batch requires 0.5 g of FeCl_3_ to process 5 pieces of fabric. For 1 m^2^, this scales to 27.78 g m^−2^. FeCl_3_ bulk purchase price from Macklin is 68 RMB 500 g^−1^, or 0.136 RMB g^−1^. Assuming reuse 5 times, the effective cost is approximately 0.76 RMB m^−2^.
3. Ethanol: Each batch uses 10 mL of ethanol to process 0.018 m^2^ of fabric. For 1 m^2^, this corresponds to 555.56 mL m^−2^. The bulk purchase price for industrial-grade ethanol is approximately 5 RMB L^−1^, or 0.005 RMB mL^−1^. Assuming reuse 5 times, the effective cost is approximately 0.56 RMB m^−2^.
4. Pyrrole (Py): Each batch uses 1 mL of Py to process 0.018 m^2^ of fabric. For 1 m^1^, this corresponds to 55.56 mL m^−2^. Bulk pricing for Py is a typical price of 100 RMB kg^−1^ (0.1 RMB g^−1^) for industrial-grade packaging (25 kg per barrel). Assuming reuse 5 times, the effective cost is approximately 1.11 RMB m^−2^.

Total cost of raw materials: 27.23 RMB m^−2^.

**Energy consumption**:

Heating the sealed container to 80 °C for 150 minutes to process 5 pieces of fabric over 5 repetitions is estimated to consume 0.5 kWh m^−2^. At an electricity rate of 0.6 RMB kWh^−1^, the energy cost is approximately 0.30 RMB m^−2^.

**Final Cost Calculation**

The total cost per m^2^ is calculated as:

Total Cost = 27.23 + 0.30 = 27.53 RMB m^−2^.

In summary, scaling up production and utilizing lower-cost industrial-grade FOS or Py can reduce the cost to approximately 28 RMB m^−2^. This low cost highlights the economic feasibility of the proposed method for large-scale production.

## 1.2. Calculation of the photothermal conversion efficiency

The photothermal conversion efficiency of the cocrystal was determined according to the previous method^1-3^. Details are as follows:

Based on the total energy balance for this system:

$\sum_{i} m_{i}C_{p, i}\frac{dT}{dt}= Q_{s}- Q_{loss}$ (S1)

Where *m_i_* (0.5 g) and *C_p, i_* (1.68 J/ (g °C) are the mass and heat capacity of system components (samples and substrate), respectively. *Qs* is the photothermal heat energy input by irradiating simulated sunlight onto composite samples, and *Q*_loss_ is thermal energy lost to the surroundings. When the temperature is maximum, the system is in balance.

$Q_{s}= Q_{loss}=hS{\Delta T}_{max}$ (S2)

Where *h* is the heat transfer coefficient, *S* is the surface area of the container, ${\Delta T}_{max}$ is the maximum temperature change.

The photothermal conversion efficiency *η* is calculated from the following equation:

$\eta= \frac{hS{\Delta T}_{max}}{I (1-{10}^{-A})}$ (S3)

Where *I* is the irradiation intensity of the incident light (1 W/cm^2^) and *A* represents the absorbance of the photothermal materials at the wavelength λ.

The calculated total absorption in the samples is weighted by the air mass (AM) 1.5 solar spectrum and integrated from 280 nm (λ_min_) to 2500 nm (λ_g_) to obtain the average absorption A.

$A= \int_{\lambda_{min}}^{\lambda_{g}} A \left( \lambda\right) \frac{dI}{d\lambda} d\lambda$ (S4)

Where *dI/dλ* is the incident solar radiation intensity per unit wavelength^4^.

To get the *hS*, a dimensionless driving force temperature, θ is introduced as follows:

$\theta= \frac{T- T_{surr}}{T_{max}- T_{surr}}$ (S5)

Where *T* is the temperature of samples, *T_max_* is the maximum system temperature (81 °C), and *T_surr_* is the initial temperature (25 °C).

And a sample system time constant $\tau_{s}$

$\tau_{s}= \frac{\sum_{i} m_{i} C_{p, i}}{hS}$ (S6)

Thus

$\frac{d\theta}{dt}= \frac{1}{\tau_{s}} \frac{Q_{s}}{hS{\Delta T}_{max}}-\frac{\theta}{\tau_{s}}$ (S7)

When the light is off, *Qs* = 0, therefore

$\frac{d\theta}{dt}= - \frac{\theta}{\tau_{s}}$ (S8)

And $t= -\tau_{s}\ln\theta$

So *hS* could be calculated from the slope of cooling time vs $\ln\theta$*.*

Therefore, $\tau_{s}$ is 49 s (Figure S12b). And the photothermal conversion efficiency (*η*) of CP-FPNF-15 is 84.6 %.

## 1.3. Calculation of the photo-to-vapor conversion efficiency

The energy conversion efficiency is another indicator used to evaluate the photothermal conversion performance. It refers to the ratio between the energy transferred to the liquid under the photothermal effect and the corresponding amount of evaporation. The energy conversion efficiency is calculated by the following equation^5^:

$\eta= \frac{ṁ E_{equ}}{C_{opt}P}$ (S9)

Where *η* is energy conversion efficiency, *ṁ* is the evaporation rate under irradiation, and *P* corresponds to the solar irradiation power of one sun (1 kW m^-2^). *C_opt_* refers to the optical concentration on the absorber surface. *E_equ_* is the equivalent evaporation enthalpy of the water in CP-FPNFs. It can be calculated by evaporating water under dark conditions assuming the same energy input:

$U= E_{equ} m_{s}= E_{0} m_{0}$ (S10)

Where *m_s_* is the mass change of the samples in the dark environment, *E_0_* and *m_0_* refer to the evaporation enthalpy and mass change of bulk water under dark conditions. We obtained the equivalent water vaporization enthalpy of bulk water and water in the CP-FPNF-15 by measuring the evaporation rate under dark conditions. The CP-FPNF-15 shows a higher evaporation rate than the bulk water under the dark condition, indicating that the CP-FPNF-15 has a lower equivalent water vaporization enthalpy of about 1235 J g^-1^ (Figure S18, S19). The equivalent water vaporization enthalpy of CP-FPNF-15 is 50% lower than that of bulk water, and its theoretical value is 2450 J g^-1^. The results explain the high evaporation rate of our fabric-based CP-FPNF-15 and suggest that reducing the water vaporization enthalpy is an effective strategy to increase the evaporation rate of solar steam generators. The calculated energy conversion efficiency of CP-FPNF-15 is 139.2%, including both solar and environmental evaporation.

## 1.4. Analysis of heat transfer mechanisms in the solar interfacial evaporation system

The heat transfer in a solar evaporation system primarily occurs through three modes: convection, conduction, and radiation.

**1.4.1. Convection**

Convective heat loss happens as energy moves from the heat source to a cooler fluid mass, following Newton's law of cooling:

$Q_{1}=h A_{s} (T_{body}-T_{c})$ (S11)

Where *Q_1_* is the heat energy, *h* is the convective heat transfer coefficient (5 W m^-2^ K^-1^), *As* represents the surface area, *T_body_* is the temperature of the material layer heaters (CP-FPNFs), and *T_c_* is the ambient fluid temperature. According to this equation, the heat loss due to convection is estimated to be approximately 1.31 %.

**1.4.2. Radiation**

Thermal radiation involves electromagnetic radiation from all objects, occurring without a transfer medium. Each object simultaneously emits and absorbs radiation, with net heat loss depending on temperature and emissivity relative to its surroundings. Radiation loss is determined by the Stefan-Boltzmann law:

$Q_{2}= \varepsilon\sigma A_{s} (T_{1}^{4}- T_{2}^{4})$ (S12)

Where *Q_2_* is the radiative heat flux, *ε* is the object's emissivity (taken here as 0.95), *σ* is the Stefan-Boltzmann constant (5.67×10^-8^ W m^-2^ K^-4^), *A_s_* is the radiating surface area, *T_1_* is the CP-FPNFs surface temperature, and *T_2_* is the temperature of the surrounding environment. The resulting thermal radiation loss is calculated at about 1.20%.

**1.4.3. Conduction**

Heat conduction occurs when thermal energy transfers from a higher temperature source to a cooler body. This can be expressed as:

$Q_{3}=C m (T_{1}- T_{2})$ (S13)

Where *Q_3_* is the heat energy, *C* is the specific heat capacity of water (4200 J kg^-1^ ℃^-1^), *m* is the mass of water, and *T_1_* and *T_2_* represent the initial and final average water temperatures after 30 minutes of solar exposure under one sun intensity. Calculations indicate that conduction loss is approximately 0.70%.

# 2. Figures (S1-S26)

## 2.1. Synthesis scheme of CP-FPNFs

**Figure S1.** Schematic of the preparation process of CP-FPNF-based Janus evaporator.

## 2.2. Structure characterization of multifunction fabrics

**Figure S2.** FTIR spectra of the nylon fabric (NF), UCP-PFNF, and CP-FPNF. The pristine NF presents characteristic peaks at 1635 cm^−1^ (-C=O stretching) and 1532 cm^−1^ (amide groups). A weak band at 1719 cm^-1^, attributing to carbonyl in over-oxidized CP-FPNF suggests the concentration of this defect in CP-FPNF is higher than that in UCP-PFNF.

**Figure S3.** The thermal image of a butterfly specimen exposed to 1 sun radiation. Leveraging multiple internal reflections, the butterfly wing structure effectively absorbs sunlight across a wide range of wavelengths, resulting in a rapid temperature increase under 1 sun irradiation.

**Figure S4.** UV absorption spectra of eluted oligomers collected on washing.

## 2.3. Microscopic surface morphology of fibers

**Figure S5.** SEM image of UCP-PFNF.

**Figure S6.** SEM images of (a) nylon fibers, (b) FOS-Fe^3+^ coated nylon fibers, and (c) CP-FPNF fiber surface.

**Figure S7.** Schematic comparison of (a) UCP and (b) CP pathways and dopant environments.

**Figure S8.** Raman spectra of CP-FPNF-0, CP-FPNF-5, CP-FPNF-10, and CP-FPNF-15.

## 2.4. Microscopic surface morphology of PPy and PFOS-PPy coatings

**Figure S9.** SEM images of the PFOS-PPy coating obtained through (a-d) solution polymerization and (e) PPy, (f) PFOS-PPy-5, (g) PFOS-PPy-10, (h) PFOS-PPy-10 scraped off from glass slides using the same confined polymerization.

## 2.5. Structure characterization of PPy and PFOS-PPy coatings

**Figure S10.** FTIR spectra of PPy, PFOS-PPy-5, PFOS-PPy-10, and PFOS-PPy-15 (with peaks at 1632 cm^-1^,1556 cm^-1^ and 1397 cm^-1^ relating to the C-C/C=C stretching vibration, vibration of pyrrole ring, and C-N stretching vibration, respectively. Peaks at 1146 cm^-1^ were attributed to C-F).^[6]^

**Figure S11.** Mott-Schottky curves of (a) PPy, (b) PFOS-PPy-5, (c) PFOS-PPy-10, and (d) PFOS-PPy-15. The flat potential values for PPy, PFOS-PPy-5, PFOS-PPy-10, and PFOS-PPy-15 were recorded at -1.53 V, -1.23 V, -0.75 V, and -0.81 V, respectively. The corresponding conduction potentials were found to be -0.93 V, -0.63 V, -0.15 V, and -0.21 V (vs. RHE, pH = 6.8). Combining the optical bandgap with the equation E_g_ = E_VB_-E_CB_, the valence band potentials were calculated to be 1.85 V, 1.38 V, 1.3 V, and 1.02 V, for PPy, PFOS-PPy-5, PFOS-PPy-10, and PFOS-PPy-15, respectively.

## 2.6. Solar evaporation performance testing

**Figure S12.** (a) The cooling curve of CP-FPNF-15 after the 1 solar irradiation and (b) the corresponding time-Inθ linear curve.

**Figure S13.** Photothermal conversion efficiency of CP-FPNF-5 and CP-FPNF-10.

**Figure S14.** Photoluminescence (PL) spectra of the samples under excitation at 295 nm. PL measurements were performed on powder samples obtained by scraping the photothermal coating from glass substrates and dispersing it in ethanol to reduce aggregation-induced quenching. A weak emission peak around 330 nm was observed, with decreasing intensity as FOS content increased, indicating enhanced non-radiative recombination.

**Figure S15.** Photothermal cycling stability of the CP-FPNF-15 under repeated light on and off irradiation.

**Figure S16.** SEM images of the CP-FPNF-15 fiber surface before and after six heating-cooling cycles. The coating remains intact with no visible damage, confirming excellent structural integrity under thermal cycling.

**Figure S17.** Photographs and contact angles of UCP-PFNF and CP-FPNFs.

**Figure S18.** The dark experiment of the bulk water and CP-FPNF-15 fabric.

**Figure S19.** Comparison of evaporation rate in the dark environment and equivalent vaporization enthalpy between bulk water and CP-FPNF-15 fabric. The red star points represent the evaporation rate and the blue columns represent equivalent vaporization enthalpy.

**Figure S20.** Evaporation rates of CP-FPNF-15-based Janus evaporator in saline water of varying concentrations.

**Figure S21.** Evaporation rates of three independently prepared CP-FPNF-15 samples at sizes up to 22×16 cm^2^ under simulated saltwater evaporation conditions (1 sun irradiation, 40% humidity). Each value represents the mean ± standard deviation of three independent measurements. Although minor fluctuations were observed, the three batches exhibited comparable average performance, confirming the repeatability of the CP strategy.

**Figure S22.** Photographs and infrared thermal image of a 22 × 16 cm^2^ CP-FPNF-15 sample under outdoor irradiation. The temperature distribution across the surface is uniform, indicating consistent photothermal performance.

**Figure S23.** Evaporation rates under varying humidity conditions (40%, 60%, 80%) controlled using a humidifier. The inset shows the humidifier used in the experiment. Each value represents the mean ± standard deviation of three independent measurements.

**Figure S24.** Conductivity evaluation of collected water purified by the CP-FPNF-15-based Janus evaporator from different water bodies using a multimeter with a constant distance between electrodes.

**Figure S25.** Comparison of 1-hour evaporation rate before and after mechanical treatment, confirming negligible performance degradation after repeated folding (Inset are photos of the CP-FPNF-15 during manual folding and rubbing). Each value represents the mean ± standard deviation of three independent measurements.

**Figure S26.** Tensile stress-strain curves of UCP-PFNF and CP-FPNF-15 fabrics, showing enhanced ductility in the latter.

# 3. Tables (S1-S3)

Table S1**. Atomic ratios of samples with different etching times in the XPS characterization**

|  | C (%) | N (%) | O (%) | Si (%) | F (%) |
| --- | --- | --- | --- | --- | --- |
| 0 s | 30.44 ± 0.99 | 0.72 ± 0.01 | 6.57 ± 0.03 | 4.75 ± 0.01 | 57.52 ± 1.52 |
| 10 s | 51.94 ± 1.13 | 1.57 ± 0.03 | 8.04 ± 0.04 | 5.71 ± 0.06 | 32.74 ± 1.42 |
| 20 s | 60.42 ± 1.32 | 1.45 ± 0.02 | 6.45 ± 0.09 | 4.25 ± 0.04 | 27.43 ± 2.03 |
| 30 s | 64.15 ± 1.21 | 1.96 ± 0.03 | 5.63 ± 0.06 | 3.76 ± 0.08 | 24.50 ± 2.78 |
| 60 s | 68.23 ± 0.08 | 1.74 ± 0.04 | 3.31 ± 0.09 | 2.55 ± 0.05 | 24.17 ± 1.99 |
| 90 s | 70.35 ± 2.45 | 2.17 ± 0.08 | 3.28 ± 0.07 | 2.27 ± 0.02 | 21.75 ± 1.61 |
| 120 s | 72.06 ± 1.57 | 2.29 ± 0.07 | 3.10 ± 0.08 | 1.94 ± 0.03 | 20.61 ± 1.52 |

**Table S2. Atomic ratios of different samples were determined by the XPS at the etching times of 0 s.**

|  | C (%) | O (%) | N (%) | F (%) | Si (%) |
| --- | --- | --- | --- | --- | --- |
| UCP-PFNF | 42.23 | 12.46 | 3.05 | 36.37 | 5.89 |
| CP-FPNF-5 | 34.07 | 6.78 | 1.21 | 53.61 | 4.33 |
| CP-FPNF-10 | 32.35 | 6.42 | 0.70 | 55.64 | 4.89 |
| CP-FPNF-15 | 31.62 | 7.12 | 0.65 | 56.14 | 4.47 |

**Table S3. Performance of various reported evaporators.**

| Material | Evaporation rate (kg m^-2^ h^-1^) | Salt concentration (wt%) | Reference |
| --- | --- | --- | --- |
| PANI-cotton fabric | 1.94 | 3.5 | Ref. ^[7]^ |
| HNG/PVA-PPy | 3.20 | 0 | Ref. ^[8]^ |
| B-SPH | 3.45 | 0 | Ref. ^[9]^ |
| PPy-coated cotton fabric | 1.54 | 0 | Ref. ^[10]^ |
| W-cotton cloth-NCC | 1.52 | 26.4 | Ref. ^[11]^ |
| PPy/PVA-F4 | 1.68 | 0 | Ref. ^[12]^ |
| Janus-SSG | 2.21 | 0 | Ref. ^[13]^ |
| CB@SBS/cotton fabric | 1.37 | 3.5 | Ref. ^[14]^ |
| Wood/ZIF-8@PDA | 2.70 | 0 | Ref. ^[15]^ |
| h-LAH/PVA-PPy | 3.60 | 0 | Ref. ^[5]^ |
| CB/PAN membrane | 1.20 | 20.0 | Ref. ^[16]^ |
| PAN@CoMn-LDH | 2.74 | 10.0 | Ref. ^[17]^ |
| PAN@CoMn-LDH | 2.93 | 7.0 |  |
| SR-3mm | 1.31 | 20.0 | Ref. ^[18]^ |
| SR-3mm | 1.21 | 25.0 |  |
| PPy/CA | 3.16 | 0 | Ref. ^[19]^ |
| La_0.9_Sr_0.1_CoO_3_/Ti_3_C_2_ | 1.40 | 3.5 | Ref. ^[20]^ |
| MXene LSCI/NF-CA | 1.60 | 3.5 | Ref. ^[21]^ |
| LB_5_O_3_ | 2.10 | 0 | Ref. ^[22]^ |
| MF/CNTs-1 | 2.23 | 0 | Ref. ^[23]^ |
| This work | 3.84 | 0 | / |

# 4. Supplementary references

[1] B. Kim, H. Shin, T. Park, H. Lim, E. Kim. NIR-sensitive poly (3, 4-ethylenedioxyselenophene) derivatives for transparent photo-thermo-electric converters. *Adv. Mater.* **2013**, *25*, 5483.

[2] B. Tang, W. L. Li, Y. Chang, et al. A supramolecular radical dimer: high-efficiency NIR-II photothermal conversion and therapy. *Angew. Chem. Int. Ed.* **2019**, *58*, 15526.

[3] Y. Wang, W. Zhu, W. Du, X. Liu, X. Zhang, H. Dong, W. Hu. Cocrystals strategy towards materials for near-infrared photothermal conversion and imaging. *Angew. Chem. Int. Ed.* **2018**, *57*, 3963.

[4] D. Zhou, R. Biswas. Photonic crystal enhanced light-trapping in thin film solar cells. *J. Appl. Phys* **2008**, *103*, 093102.

[5] X. Zhou, F. Zhao, Y. Guo, B. Rosenberger, G. Yu. Architecting highly hydratable polymer networks to tune the water state for solar water purification. *Sci. Adv.* **2019**, *5*, eaaw5484.

[6] Y. Li, S. Yan, X. Jia, et al. Uncovering the origin of full-spectrum visible-light-responsive polypyrrole supramolecular photocatalysts. *Appl. Catal. B: Environ.* **2021**, *287*, 119926.

[7] Z. Liu, B. Wu, B. Zhu, Z. Chen, M. Zhu, X. Liu. Continuously producing watersteam and concentrated brine from seawater by hanging photothermal fabrics under sunlight. *Adv. Funct. Mater.* **2019**, *29*, 1905485.

[8] F. Zhao, X. Zhou, Y. Shi, et al. Highly efficient solar vapour generation via hierarchically nanostructured gels. *Nat. Nanotechnol.* **2018**, *13*, 489.

[9] C. Lei, J. Park, W. Guan, Y. Zhao, K. P. Johnston, G. Yu. Biomimetically assembled sponge-like hydrogels for efficient solar water purification. *Adv. Funct. Mater.* **2023**, *33*, 2303883.

[10] Y. Xu, T. Xu, Y. Guo, W. Liu, J. Wang. Scalable and biomimetic anti-oil-fouling photothermal fabric for efficient solar-driven interfacial evaporation. *Sep. Purif. Technol.* **2023**, *312*, 123289.

[11] Z. Qin, H. Sun, Y. Tang, S. Yin, L. Yang, M. Xu, Z. Liu. Bioinspired hydrophilic-hydrophobic Janus composites for highly efficient solar steam generation. *ACS Appl. Mater. Interfaces* **2021**, *13*, 19467.

[12] B. Wen, X. Zhang, Y. Yan, et al. Tailoring polypyrrole-based Janus aerogel for efficient and stable solar steam generation. *Desalination* **2021**, *516*, 115228.

[13] H. Yao, P. Zhang, C. Yang, et al. Janus-interface engineering boosting solar steam towards high-efficiency water collection. *Energy Environ. Sci.* **2021**, *14*, 5330.

[14] Y. Tian, Y. Li, X. Zhang, et al. Breath-figure self-assembled low-cost Janus fabrics for highly efficient and stable solar desalination. *Adv. Funct. Mater.* **2022**, *32*, 2113258.

[15] Y. Lu, D. Fan, Z. Shen, H. Zhang, H. Xu, X. Yang. Design and performance boost of a MOF-functionalized-wood solar evaporator through tuning the hydrogen-bonding interactions. *Nano Energy* **2022**, *95*, 107016.

[16] W. Xu, X. Hu, S. Zhuang, et al. Flexible and salt resistant Janus absorbers by electrospinning for stable and efficient solar desalination. *Adv. Energy Mater.* **2018**, *8*, 1702884.

[17] R. Zheng, T. Lin, W.-L. Zhao, et al. Hierarchical CoMn-LDH based photothermal membrane with low evaporation enthalpy and narrow bandgap toward highly efficient solar-driven evaporation. *Chem. Eng. J.* **2023**, *470*, 144103.

[18] C. Dang, H. Wang, Y. Cao, et al. Ultra salt-resistant solar desalination system via large-scale easy assembly of microstructural units. *Energy Environ. Sci.* **2022**, *15*, 5405.

[19] Y. Ren, R. Zhou, T. G. Dong, Q. Lu. Wood-inspired polypyrrole/cellulose aerogels with vertically aligned channels prepared by facile freeze-casting for efficient interfacial solar evaporation. *J. Mater. Chem. A* **2023**, *11*, 17748.

[20] H. Zhang, Y. Lu, D. Fan, X. Xu, X. Li, X. Yang. Multifunctional Ti_3_C_2_ decorated perovskite La_1-x_Sr_x_CoO_3_ nanorods for efficient energy conversion. *Chem. Commun.* **2023**, *59*, 2986.

[21] H. Zhang, X. Xu, G. Zhang, J. Yang, Y. Lu. Surface hydration of trimethylamine N-oxide/perovskites hybrid aerogel facilitates anti-fouling solar desalination and seawater electrocatalysis. *Colloid. surface. A* **2024**, *695*, 134027.

[22] Y. Lu, Z. Li, J. Liang, X. Xu, G. Zhang, H. Min. Vermiculite-derived MgFe nanosheet-enhanced hydrogel with dual photothermal and photocatalytic Fenton functions for solar water purification. *Desalination* **2024**, *592*, 118196.

[23] Y. Lu, Z. Li, G. Zhang, et al. A portable device utilizing high-entropy perovskite aerogels for efficient energy conversion from atmospheric water. *Adv. Fiber Mater.* **2025**, *7*, 563.
